# A simple and robust HPLC method to analyze lutein in wheat

**DOI:** 10.1016/j.mex.2022.101926

**Published:** 2022-11-13

**Authors:** Bangbang Wu, Xiaohua Li, Xingwei Zheng, Jiajia Zhao, Ling Qiao, Jun Zheng

**Affiliations:** Institute of Wheat Research, Shanxi Agricultural University, Linfen, China

**Keywords:** Lutein, High-performance liquid chromatography, Wheat

## Abstract

A method for the determination of lutein, the main component of carotenoid in wheat by high performance liquid chromatography (HPLC) was established. The samples were extracted with acetone and methanol (acetone : methanol 7 : 3, v / v, 0.1 % BHT w / v), and fully dissolved in methanol / acetonitrile / n-hexane (7 : 2 : 1, v / v / v) mixed solution. Poroshell 120 EC-C18 column (4.6 mm× 150 mm, 4μm) was used as the separation column, acetonitrile,methanol and n-hexane solution were used as mobile phase with gradient elution, flow rate was 1.0 mL·min^−1^, column temperature was 25 ℃, injection volume was 50 μL, and detection wave length was 450 nm. The mass concentration of lutein had a good linear relationships with the chromatographic peak area in the range of 0.5-5.0 μg·mL^−1^, the correlation coefficients were all not less than 0.999 8, and the detection limits was 12 ng·mL^−1^. The recoveries rate of standard addition in sampling were 95.50%-97.33%, and the relative standard deviations of determination results were 1.20%-1.66%(n=5). The method is sensitive, precise, rapid, accurate and efficient. The advantages of this study can be summarized in the next bullet points:

• Can be used for the determination of lutein in wheat.
• The detection time is short.
• Limit of detection (LOD) and limit of quantification (LOQ) for lutein was 12 ng·mL^−1^ and 42 ng·mL^−1^.

## Graphical abstract

**Figure:**
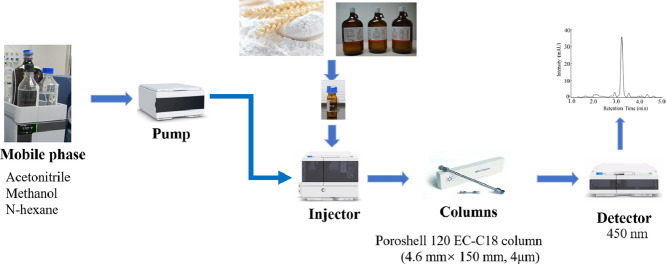

Specifications Table

**Table:** 

Subject area:	Agricultural and Biological Sciences
More specific subject area:	Analytic and sample pre-treatment
Name of your method:	Determination of lutein in Wheat grains by High performance liquid Chromatography
Name and reference of original method:	Evaluation and Genetic variation of lutein content in Chinese common wheat. Journal of Cereal Science.
Resource availability:	The data are available with this article

## Methods details

### Chemicals and reagents

A lutein standard (HPLC grade ≥95%) was purchased from the Sigma-Aldrich Chemical Co. (St. Louis, USA) and kept in darkness at 4℃. HPLC grade methanol, acetonitrile and n-hexane, and butylated hydroxytoluene (BHT, ≥ 99% FG), and acetone (GR grade) were purchased from the Shanghai Macklin Biochemical Co. HPLC-grade deionized water was sourced with a Milli-Q 50 system (Millipore Iberica S.A., Madrid, Spain). A stock solution of the lutein standard was dissolved in dicholoromethane, diluted to 0.1 mg/mL with methanol, and stored at -80℃. Lutein standards at 1.0, 1.25, 2.5, 5.0 and 10.0 μg/mL were used to generate a regression equation for quantification of test samples.

### Sample treatment

Harvested wheat grains were kept at 37℃ for 72 h, the moisture content of grain samples was determined by AACC 14-50 method [1] and maintained at approximately 14.0%, and ground into fine powder using a Brabender Quandrumat Junior mill (Brabender GmbH & Co. KG, Duisburg, Germany). The flour was stored at 18°C under low light to minimize degradation before lutein extraction.

Lutein was extracted from the flour. 1.0 g of whole wheat flour was added to 6 mL of extraction solution (acetone: methanol 7:3, v/v, 0.1% BHT w/v) in either polypropylene or borosilicate tubes and mixed vigorously in a vortex mixer for 45 s. The mixture was shaken at 80 rpm for 1 h at 35°C on a constant temperature shaker under low light. Extracts were centrifuged for 10 min at 10,528 rcf (4°C). The solid extraction residue was added to 6 mL of extraction solution, shaken, and centrifuged. The combined supernatants were evaporated to dryness at 35°C in a nitrogen stream evaporator.

The dry residue was reconstituted in 3 mL diethyl ether and 3 mL of the hydrolysis medium (50% aqueous KOH and ethanol 1:9, v/v) and shaken for 2 hours at room temperature in darkness. Three mL of the hydrolysate were then transferred into another falcon tube and 3 mL diethyl ether/ n-hexane (1:1, v/v) and 3 mL water were added. Tubes were centrifuged at 6738 rcf for 10 min at 4°C and the lower (aqueous) phase containing KOH was removed with a needle and syringe. The organic layer was evaporated to dryness at 35°C in a nitrogen stream evaporator.

The dry residue was added to 0.5 mL acetonitrile: methanol: n-hexane (7:2:1, v/v/v), transferred to 1.5 mL tubes, and centrifuged for 5 min at 13,225 rcf (4°C). The supernatants were filtered through 0.22 μm PVDF spring filters and stored in brown micro-centrifuge tubes at -20°C until HPLC analysis. For automatic sampling during HPLC analysis, 50 μL samples were transferred into amber glass vials with screw caps.

### HPLC analysis

Chromatographic analysis was performed on an Agilent 1260 HPLC system (Palo Alto, CA) using an InfinityLab Poroshell 120EC-C18 column (4.6*150 mm, 4 μm). An injection volume of 50 μL and a flow rate of 1 mL/min were used. The temperature of the injector and column oven were maintained at 4°C and 25°C, respectively. Lutein was detected at 450 nm. The mobile phases were acetonitrile (phase A), methanol (phase B), and n-hexane (phase C). The mobile phase was first degassed by sonication for 20 min. The gradient was programmed as follows: initial conditions of 76% solution A, 21.5% solution B, 2.5% solution C. The proportion of mobile phase A decreased linearly from 76% to 70% over 20-22 min and B decreased linearly from 21.5% to 20% over 20-22 min. In the following 28-30 min, phase A increased to 76% and phase B increased to 21.5%.

Five different dilutions of the lutein standard were used to set the analysis conditions and determine the retention time. Lutein in wheat samples was identified by retention time and quantified using a five-point calibration curve regression line generated with the external standards. The linearity of the calibration lines was given. Accuracy was evaluated by spiking lutein standard, then the recovery percentage of the added concentrations of the lutein was evaluated.

### Method validation

**Fig. 1:**
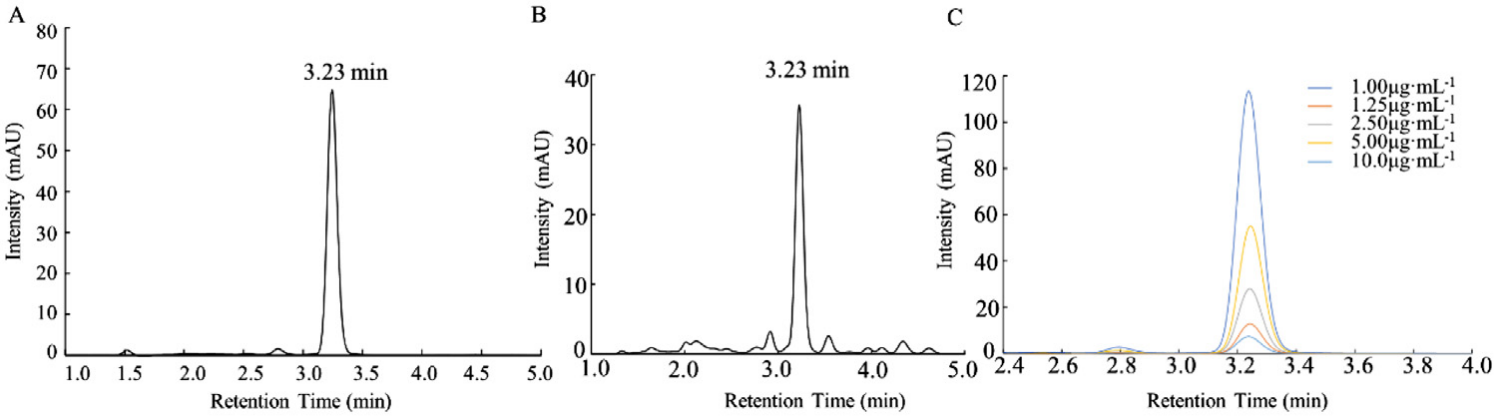
HPLC chromatogram of lutein from standard(A),wheat samples(B),lutein with different concentrations(C)

According to the above chromatographic conditions, the retention time of standard sample was 3.23 min, and the retention time of lutein in wheat sample was 3.23 min, indicating that the retention time of wheat sample was consistent with that of standard sample, and the same substance was detected (Fig. 1. A B). Five different concentrations of standard solution were used to make the standard curve, and the repeatability of the determination method of wheat lutein under the determined elution conditions was verified (Fig. 1. C). The five standard curves were single peak at 450 nm with good retention time, repeatability and relatively stable, which indicated that the method for the determination of lutein was reliable.

To test the robustness of extraction and analytical method, recovery experiments were investigated. Four different accessions were tested, which are within the range of quantification (above LOQ), the average recovery rate was above 95% (Table 1).

**Table 1:** The spiked recoveries of lutein content with wheat genotypes

Sample	Sample contents (μg·g^−1^)	Added (μg)	Detection (μg·g^−1^)	Recoveries (%)
1	1.22	1.5	2.68	97.33
2	2.36	2.5	4.76	96.00
3	1.85	2	3.76	95.50
4	2.38	2.5	4.81	97.20

The reproducibility, presented as relative standard deviation, between 3.25 – 4.38% was satisfying over the entire range of concentrations tested (Table 2).

**Table 2:** Relative standard of lutein content with wheat genotypes (n=5)

Sample	Samples contents (μg·g^−1^)					Average (μg·g^−1^)	RSD/%
	1	2	3	4	5		
1	1.34	1.37	1.32	1.25	1.28	1.31	3.25
2	2.76	2.64	2.93	2.99	2.85	2.83	4.38
3	1.89	1.97	1.95	1.74	1.85	1.88	4.36
4	2.53	2.66	2.37	2.49	2.59	2.53	3.86

The method was validated by calculating the limit of detection (LOD) and limit of quantification (LOQ). The limit of detection and quantitative are calculated by S/N≥3 and S/N≥10, respectively (Table 3).

**Table 3:** Summary of method validation parameters

Substance	Linear range	Linear regression	LOD	LOQ
	(μg mL^−1^)	(R^2^)	(ng mL^−1^)	(ng mL^−1^)
Lutein	1.0-10.0	0.9998	12	42

## Data Availability

Data will be made available on request.
